# Diverse Roles of Neurotensin Agonists in the Central Nervous System

**DOI:** 10.3389/fendo.2013.00036

**Published:** 2013-03-22

**Authors:** Mona Boules, Zhimin Li, Kristin Smith, Paul Fredrickson, Elliott Richelson

**Affiliations:** ^1^Neuropsychopharmacology Laboratory, Department of Neuroscience, Mayo Clinic FloridaJacksonville, FL, USA

**Keywords:** neurotensin, endocrinology, schizophrenia, pain, psychostimulant abuse, central nervous system

## Abstract

Neurotensin (NT) is a tridecapeptide that is found in the central nervous system (CNS) and the gastrointestinal tract. NT behaves as a neurotransmitter in the brain and as a hormone in the gut. Additionally, NT acts as a neuromodulator to several neurotransmitter systems including dopaminergic, sertonergic, GABAergic, glutamatergic, and cholinergic systems. Due to its association with such a wide variety of neurotransmitters, NT has been implicated in the pathophysiology of several CNS disorders such as schizophrenia, drug abuse, Parkinson’s disease (PD), pain, central control of blood pressure, eating disorders, as well as, cancer and inflammation. The present review will focus on the role that NT and its analogs play in schizophrenia, endocrine function, pain, psychostimulant abuse, and PD.

## Introduction

Neurotensin (NT) is a 13 amino acid neuropeptide (*p*Glu-Leu-Tyr-Glu-Asn-Lys-Pro-Arg-Arg-Pro-Tyr-Ile-Leu) that was first isolated from bovine hypothalamus by Carraway and Leeman (1973). It is abundant in gastrointestinal tract where it plays a role in gut motility. NT has also been detected in peripheral organs including: heart, liver, lung, pancreas, spleen, and small intestine. It is a paracrine and endocrine modulator of the cardiovascular system and of the digestive tract and acts as a growth factor on a variety of normal and cancerous cells. In addition, NT is widely distributed in the central nervous system (CNS), with the highest concentration in the amygdala, lateral septum, ventral tegmental area (VTA), and substantia nigra (SN).

In brain, NT plays a role in naloxone-independent antinociception (Clineschmidt et al., 1979), hypothermia (Bissette et al., 1976), control of anterior pituitary hormone secretion, and muscle relaxation (Kitabgi et al., 1992). NT also controls central blood pressure, and inflammation (St-Gelais et al., 2006 for review). The physiological functions of NT have been recently reviewed (Mustain et al., 2011).

Many literature reviews have presented the basic role of brain NT through its relationship with dopaminergic mesotelencephalic projection system and its modulating glutamatergic transmission and other signals (Seutin, 2005; Boules et al., 2006; Antonelli et al., 2007; Ferraro et al., 2008). The current review discusses the potential clinical function of NT and the potential clinical use of its analogs as novel therapy for neuropsychiatric disorders such as schizophrenia, addiction, Parkinson’s disease (PD), and pain.

## Neurotensin Receptors

Neurotensin mediates its effects through three NT receptors (NTRs), the high affinity NTS1, the low affinity NTS2, and NTS3. Both NTS1 and NTS2 are G-protein coupled receptors, with seven-transmembrane domain. NTS1, the most studied NTR, is expressed in both neurons and glial cells and is broadly distributed in the CNS including medial septal nucleus, nucleus basalis magnocellularis, suprachiasmatic nucleus, SN, and VTA (Elde et al., 1990) as well as small dorsal root ganglion neurons of the spinal cord (Zhang et al., 1995). Activation of NTS1 induces excitatory effect through G-proteins, resulting in intracellular calcium influx. In turn, there is increased intracellular levels of cAMP, cGMP, and IP3; and increased activities of PLC, PKC, and Na+/K+-ATPase (Gilbert and Richelson, 1984; Watson et al., 1992; Yamada and Richelson, 1993; Hermans et al., 1994; Slusher et al., 1994; Poinot-Chazel et al., 1996; Lopez Ordieres and Rodriguez de Lores Arnaiz, 2000; Trudeau, 2000). NTS1 has a close association with dopaminergic and glutamatergic systems.

A high density of NTS1 receptors is co-localized with dopamine (DA) neurons in the ventral mesencephalon (Brouard et al., 1992; Nicot et al., 1995; Boudin et al., 1998). NT antagonizes DA effects at D2 receptors via NTS1/D2 receptor–receptor interaction (Jiang et al., 1994; Farkas et al., 1996). Several studies suggested that NTS1 receptors on mesencephalic DA neurons play an important role in sensitization to psychostimulant drugs and drug addiction (Binder et al., 2001a; Berod and Rostene, 2002; Panayi et al., 2005). Our recent work shows that (1) NTS1 knockout mice (NTS1^−/−^) mice are spontaneously hyperactive and more sensitive to d-amphetamine-induced hyperactivity (Liang et al., 2010); (2) NTS1^−/−^ mice have higher basal levels of DA and higher levels of amphetamine-induced DA release in striatum as compared with results for WT mice (Liang et al., 2010); and (3) NTS1^−/−^ have abnormal D2/D1 ratio in striatum (Liang et al., 2010), leading to a decrease of d-amphetamine-induced glutamate and GABA release in striatum (Li et al., 2010b). The results suggest that NTS1 through possible modulation of DA receptor plays an important role in the dysregulation of striatal DA function which is thought to be secondary to a glutamate deficiency in schizophrenia through possible modulation of DA receptor. Additionally, a possible interaction between NTS1 and D1 receptor in medial prefrontal cortex (mPFC) has been proposed in one of our recent studies (Li et al., 2010c). The same work also shows that there are significantly lower basal glutamate levels and lower density of *N*-methyl-d-aspartate (NMDA) receptor 2A subunit in the mPFC of NTS1 null mice as compared with results for WT mice (Li et al., 2010c). These data are consistent with the hypothesis that NTS1 is involved with the pathophysiology of the hypofunction of the glutamatergic system in schizophrenia.

The relationship between NTS1 and glutamatergic system has been investigated by Antonelli’s group for years. Activation of NTS1 promotes and reinforces endogenous glutamate signaling in discrete brain regions by increasing the activation of PKC and leading to phosphorylation of the NMDA receptor. NTS1 receptors are highly expressed in nigro-striatal dopaminergic neurons, which degenerate in PD. It has been hypothesized (Antonelli et al., 2007) that in Parkinson’s patients, possible elevated NT levels in the basal ganglia cause the NTS1 enhancement of excitotoxic glutamate signaling contributing to the DA neuron neurodegeneration in SN. However, paradoxically, NT or an NTS1 agonist has antiparkinson-like effect (Jolicoeur et al., 1991; Boules et al., 2001b). The discrepancy is likely due to different routes and time course of administration: local infusion in the striatum or SN for 60 min versus acute systemic i.p. administration. We have discussed previously that the effects of a NTR agonist given outside the brain cannot be predicted from studies in which a NTR agonist is injected into discrete areas of the brain (Richelson et al., 2003). Both groups agree that there is a critical role for NTS1 in PD.

NTS2 is localized mainly in the olfactory system, the cerebral and cerebellar cortices, the hippocampal formation, and selective hypothalamic nuclei, VTA, and SN. Importantly, strong expression of NTS2 receptors is in the areas related to pain, namely, the periaqueductal gray (PAG) and the rostral ventrolateral medulla (RVLM). Local administration of NT into the PAG and RVLM induces opioid-independent analgesia (Behbehani, 1992) through non-NTS1 dependent mechanisms (Dubuc et al., 1994). Antisense oligonucleotide knockdown of NTS2 receptor significantly decreases NT-induced analgesia, while oligodeoxynucleotides against NTS1 had no effect in this regard. These results are also supported by recent data from NT (Remaury et al., 2002) and the NTS2 receptor-selective agonist NT79 (Boules et al., 2010). Taken together it suggests that NTS2 plays an important role in pain.

NTS3/sortilin is a single transmembrane amino acid receptor, structurally unrelated to either NTS1 or NTS2. Its mRNA is expressed throughout the brain with high levels of expression in the SN, hippocampal formation, supraoptic nucleus, piriform and cerebral cortices, and medial and lateral septal nuclei (Mazella, 2001; Sarret et al., 2003). NTS3/sortilin participates in the modulation of NT intracellular sorting (hence the name “sortilin”) and signaling processes (Sarret et al., 2003) and has been associated with the phosphatidylinositol 3-kinase and mitogen-activated protein kinase pathways in glial cells (Martin et al., 2003). NTS3/sortilin has been implicated in cell death in responsive cells including neurons. This is thought to be due to the ability of NTS3/sortilin to bind the unprocessed form of nerve growth factor (proNGF) (Nykjaer et al., 2004). Additionally, SorLA/LR11, a mosaic protein of the vacuolar protein sorting 10 protein (Vps10p) domain receptor family and the low density lipoprotein receptor (LDLR) family, represents the fourth NTR (NTS4). It has a similar structure as NTS3/sortilin, with a wide distribution in the brain, especially in the hippocampus, cerebellum, cingulate gyrus, entorhinal cortex, red nucleus, and oculomotor nucleus (Motoi et al., 1999). NTS4 may be involved in the intracellular trafficking and in termination of NT signaling (Jacobsen et al., 2001).

## NT and Neurotransmitter Systems

Neurotensin acts as a primary neurotransmitter as well as a modulator of other neurotransmitter systems such as the dopaminergic, glutamatergic, GABAergic, cholinergic, and serotonergic systems (Rakovska et al., 1998; Ferraro et al., 2008; Petkova-Kirova et al., 2008). The functional and anatomical NT/DA interactions have been the most extensively studied and reviewed (Binder et al., 2001a; Jomphe et al., 2006; Fawaz et al., 2009; Thibault et al., 2011; Tanganelli et al., 2012).

### Neurotensin and dopamine

As mentioned in the previous section, NT and DA are co-localized in a subpopulation of mesencephalic neuron within the VTA (Hokfelt et al., 1984; Seroogy et al., 1988; Bean and Roth, 1992). Additionally, NT-like immunoreactivity is highly expressed in areas enriched with DA cell bodies and nerve terminals such as SN, VTA, neostriatum, and nucleus accumbens (NA) (Quirion et al., 1982). NT also forms synaptic contacts with a subpopulation of tyrosine hydroxylase (TH) immunoreactive neurons (Woulfe and Beaudet, 1989). These data strongly indicate a modulatory function of NT on DA neurotransmission.

Neurotensin directly or indirectly (e.g., through glutamate release) modulates DA neurotransmission. NT can modulate DA through: (1) up-regulating TH gene expression (Burgevin et al., 1992a,b) or (2) decreasing the DA binding affinity for the DA D_2_ receptors (Fuxe et al., 1992a; Li et al., 1995). NT also opposes DA D_2_ receptor agonist-induced auto-inhibition of DA cell firing (Shi and Bunney, 1992). Allosteric receptor–receptor interactions between NT and DA D_2_ receptors, as well as second messenger-dependent receptor alterations, such as phosphorylation and dephosphorylation, have also been implicated (see Fuxe et al., 1992b for review). It is important to mention that within the terminal fields, NT opposes the effects of DA both pre- and post-synaptically, leading either to an increase or to a decrease in DA transmission, depending on the intrinsic anatomical location of NTRs (for a more comprehensive review of the modulatory effects of NT on DA neurotransmission, we refer the reader to Binder et al. (2001a) and Kinkead and Nemeroff (2004).

### NT and serotonin

Neurotensin receptors are present on serotonergic neurons in the nucleus raphe magnus and dorsal raphe, where NT causes an increase in their firing rates (Jolas and Aghajanian, 1996; Li et al., 2001). Therefore, NT has been proposed to play a role in functions known to be affected by the serotonergic system including antinociception (Buhler et al., 2005; Boules et al., 2010), sleep-wake cycle (Jolas and Aghajanian, 1997), and stress (Corley et al., 2002).

### NT and glutamate

There has been controversy regarding the effect of NT on glutamate release and modification of the glutamatergic and NMDA receptors. NT increases glutamate release in the striatum, globus pallidus, frontal cortex, and SN (Ferraro et al., 2011, 2012) implicating NT in conditions such as stroke, Alzheimer’s disease, and PD.

Others have reported that systemic administration of NT analogs blocks ethanol-induced increases in glutamate levels in the striatum (Li et al., 2011), and decreases phencyclidine (PCP)-induced increases in glutamate in the prefrontal cortex (Li et al., 2010a). These data support the idea that the antipsychotic-like effects of NT may be mediated in part by its modulatory effect on glutamate.

## NT and Hypothalamic-Pituitary Axis

### Effect of NT on hormone release

The presence of NT in the anterior pituitary gland and hypothalamus and its storage and release at the median eminence implicate NT and its receptors in neuroendocrine regulation (Rostene and Alexander, 1997). NT is thought to possess a paracrine or an autocrine role within the hypothalamus and the anterior pituitary (Bachelet et al., 1997; Bello et al., 2004). NT stimulates both directly and indirectly, the synthesis of corticotrophin releasing hormone (CRH), gonadotropin-releasing hormone (GnRH) (Cooke et al., 2009), growth hormone-releasing hormone (GHRH) (Blackburn et al., 1980), and prolactin secretion at anterior pituitary and median eminence (Memo et al., 1986).

#### CRH-ACTH

Neurotensin stimulates the activity of the hypothalamic-pituitary CRH-adrenocorticotropin hormone (ACTH) system. Central administration of NT increases ACTH and corticosterone in the presence of CRH receptor activation in the paraventricular nucleus (PVN) (Nussdorfer et al., 1992; Rowe et al., 1995). The modulatory action of NT on the pituitary-adrenocortical function in rats has been reported to be biphasic. The lower dose of NT exerts a stimulatory effect while the higher dose appears to have an inhibitory effect on both the pituitary-ACTH release and adrenocortical secretion (Malendowicz and Nussdorfer, 1994). NT also enhances the release of both CRH-ir and ACTH-ir in rat adrenal medulla (Mazzocchi et al., 1997).

#### Gonadotropic hormones

Many neurons in the anteroventral periventricular (AVPV) and medial preoptic nuclei (MPN) express estrogen receptors and project to GnRH neurons (Smith and Wise, 2001). Surprisingly, GnRH neurons do not seem to possess intracellular estrogen receptors (Herbison and Theodosis, 1993) and evidence suggests that other neurons mediate the stimulatory effects of estrogen on GnRH secretion (Shivers et al., 1983). Interestingly, GnRH neurons were found to express NTS1 receptors (Herbison and Theodosis, 1992) and GABA receptors (Herbison et al., 1993). The number of GnRH neurons expressing NTS1-mRNA peaks during proestrus suggesting that NT directly stimulates GnRH neurons contributing to luteinizing hormone (LH) surge (Smith and Wise, 2001). There has been some controversy regarding the role of NT on LH surge. While some report that the administration of NT directly into the medial preoptic area (where GnRH cell bodies reside) evokes pre-ovulatory GnRH/LH surge (Alexander et al., 1989a), others indicate that NT has no effect on GnRH-stimulated LH release (Leiva and Croxatto, 1994). The former group showed that immunoneutralization of NT in the preoptic region attenuates the LH surge induced by estrogen and progesterone treatment in ovariectomized rats (Alexander et al., 1989b). The effect of NT on LH secretion requires intact dopaminergic and alpha adrenergic systems (Akema and Kimura, 1989).

#### Growth hormone

Neurotensin is co-expressed with growth hormone (GH) releasing factor in the arcuate nucleus (Niimi et al., 1991) and modulates GH release. NT regulates GH secretion, in prepubertal children and adults (Bozzola et al., 1998a), as well as in neonates (Bozzola et al., 1998b), through the modulation of somatostatin release from the median eminence. In rats, estrogen plays a facilitatory role on NT-induced GH release that is independent of hypothalamic GHRH or somatostatin release (Ibanez et al., 1993).

#### Prolactin

With respect to prolactin release, NT has opposite actions, an inhibitory effect at the hypothalamic site and an excitatory effect at the pituitary. NT elevates plasma prolactin and GH levels in both normal and estrogen-progesterone pretreated male rats (Rivier et al., 1977). The inhibitory action of NT on prolactin release is mediated by the release of DA into the hypophyseal portal vein, which delivers the neurotransmitter to the anterior pituitary gland causing inhibition of prolactin release (Vijayan et al., 1988; McCann and Vijayan, 1992; Pan et al., 1992).

#### Thyroid hormones

Neurotensin also participates in the neuroendocrine control of the thyroid hormones by regulating thyroid releasing hormone (TRH) function and thyroid stimulating hormone (TSH) secretion. These data indicate that NT is involved in the metabolic actions of these hormones. The role of NT in the hypothalamic-anterior pituitary-thyroid axis and the functional cooperation between NT and thyroid hormones are excellently reviewed by Stolakis et al. (2010).

#### Effect of NT on feeding

In addition to its presence in the CNS NT is also found in neuroendocrine cells of the small bowel. There, NT participates in enteric digestive processes, gut motility, and intestinal inflammatory mechanisms. It also plays an important role in intestinal lipid metabolism, thus controlling appetite, weight status, and food intake (Kalafatakis and Triantafyllou, 2011). Additionally, NT exerts central control on blood glucose, feeding patterns, and body weight.

Centrally administered NT reduces appetite (Luttinger et al., 1982; Hawkins, 1986; Cooke et al., 2009), an effect that is mainly mediated by NTS1 (Remaury et al., 2002; Kim et al., 2008; Kim and Mizuno, 2010).

The anorectic effect of centrally administered NT is potentiated by peripheral injection of DA agonists, l-DOPA and bromocriptine, results suggesting that the effect of NT may be mediated by its ability to increase the activity of dopaminergic neurotransmission in the CNS (Hawkins et al., 1986).

Similar effects were shown for the NT agonist, PD149163, in rats and ob/ob mice (Feifel et al., 2010a) as well as the non-selective NT agonist NT69L in Sprague-Dawley and in obese Zucker rats (Boules et al., 2000). The later study also showed that the effect of NT69L on food intake and body weight is due to functional interactions of NT with brain amines, and metabolic and endocrinological systems. NT69L transiently increases blood glucose and corticosterone levels and decreases TSH and T4 in Sprague-Dawley and in Zucker rats. NT69L also decreases norepinephrine in both the hypothalamus and NA, and increases DA, its metabolite 3,4-dihydroxyphenylacetic acid (DOPAC), and serotonin. These results indicate that feeding and energy expenditures are modulated by the interplay of hormones and neurotransmitters in the CNS (Brunetti et al., 2005).

Additionally, the effect of NT on feeding and body weight is also mediated, in part, by its action on the anorexigenic hormone leptin, which is secreted by adipocytes and regulates food intake by acting on hypothalamic neurons including NT-producing neurons (Beck et al., 1998; Sahu et al., 2001). Leptin together with insulin and α-melanocyte-stimulating hormones increase NT expression in the hypothalamus (Sahu, 1998; Cui et al., 2005). Similarly, the anorectic effect of leptin is at least partly mediated through central NTS1 and the leptin-NTS1 signaling pathway is involved in the regulation of food intake and the in the control of energy balance (Leinninger et al., 2011) since the lack of NTS1 reduces sensitivity to the anorectic action of leptin, causing hyperphagia and abnormal weight gain (Kim et al., 2008).

### Effect of hormones on NT synthesis and release in hypothalamic-pituitary axis

As NT modulates hypothalamic-pituitary axis (HPA) hormones, circulating hormones influence NT synthesis in the hypothalamus and anterior pituitary, results that suggest that NT mediates feedback effects of the hormones on neuroendocrine cells (Rostene and Alexander, 1997). Estrogen and progesterone regulate the activity of NT-synthesizing neurosecretory cells located in the hypothalamic arcuate nucleus (Alexander, 1999). Estrogen increases the secretion of NT at the median eminence and alters NT binding and NTS1-mRNA expression in the rostral preoptic nucleus (Moyse et al., 1988; Alexander, 1993; Watanobe and Takebe, 1993; Alexander and Leeman, 1994). GHRH neurosecretory cells synthesize NT under basal conditions (Niimi et al., 1991) and GH injection increases NT plasma levels in human (Schimpff et al., 1994).

Neurotensin’s endocrine activity, and its modulatory effects on several neurotransmitter systems, kindled many studies suggesting that NT plays a role in the pathophysiology of several CNS disorders including PD, schizophrenia, and psychostimulant and nicotine addiction as well as pain and eating disorders (Caceda et al., 2006).

## NT and Neuropsychiatric Disorders

### Schizophrenia

Schizophrenia is a devastating psychotic disorder that affects approximately 1% of the population worldwide. The onset of illness usually occurs relatively early in life with most patients experiencing long-lasting adverse effects accompanied by severe impairment (reviewed in Carpenter and Buchanan (1994). The disease is manifested by positive symptoms (delusions, hallucinations, and an altered perception of reality), and negative symptoms (apathy, cognitive blunting, and social withdrawal), as well as a disorganization of thought and behavior. Many schizophrenic patients have difficulty holding a job or caring for themselves, placing a significant burden on their families and society. Additionally, increased mortality is associated with schizophrenia as patients experience a 20% shorter lifespan than the general population and are at an increased risk for committing suicide (Newman and Bland, 1991; Goff et al., 2001).

The exact cause(s) for the pathophysiology and progression of schizophrenia are not known; however it is generally accepted that associated symptoms are syndromal in nature, rather than manifestations of a single disease (Carpenter et al., 1999). These symptoms are likely caused by several compounding biochemical abnormalities and influenced by both genetics and environment. Theories as to the origin of these abnormalities focus on the dopaminergic system, but have more recently been expanded to include the serotonergic, γ-aminobutyric acid-ergic, and glutamatergic systems, as well as the NT system.

One of the earliest and most studied theories is the DA hypothesis of schizophrenia (Carlsson, 1988; Toda and Abi-Dargham, 2007; Howes and Kapur, 2009). While this theory originally emphasized general hyperdopaminergia as a causative factor in schizophrenia (Snyder, 1976), more recent versions focus on the balance of DA within discreet regions of the brain. The current theory predicts that hyperactive subcortical mesolimbic DA projections (resulting in hyperstimulation of D_2_ receptors) are the main cause for positive symptoms, while hypoactive mesocortical DA projections to the PFC (resulting in hypostimulation of D_1_ receptors) are the main cause for negative symptoms and cognitive impairment (Weinberger, 1987; Toda and Abi-Dargham, 2007). There is strong evidence for a close relationship between the dopaminergic system and NT (Nemeroff, 1986; Kitabgi et al., 1989), suggesting a role for NT in the pathophysiology of schizophrenia.

#### Neurotensin and schizophrenia

A role for NT in schizophrenia has been hypothesized for over three decades (Nemeroff, 1980). Specifically, the NT system is closely associated with the dopaminergic system, and deficits in NT neurotransmission have been implicated in the pathophysiology of schizophrenia. Schizophrenics have a 40% decrease in NTRs in the entorhinal cortex (Wolf et al., 1995), and schizophrenics with decreased NT concentrations in their cerebral spinal fluid have significantly higher levels of pretreatment psychopathology (Sharma et al., 1997).

NT receptors have been detected on DA cell bodies in the SN and VTA (Palacios and Kuhar, 1981; Szigethy and Beaudet, 1989) and NTR activation has an excitatory effect on midbrain DA neurons (Pinnock, 1985; Seutin et al., 1989; Mercuri et al., 1993). NT can inhibit DA D_2_ autoreceptor function, thus relieving auto-inhibition of DA transmission (Jomphe et al., 2006) thereby increasing DA release, firing rate, and the synthesis of the rate limiting enzyme for DA synthesis, TH (Binder et al., 2001b). Because of this close association of NT with dopaminergic neurons and its neuromodulatory effects on the dopaminergic system, NT is hypothesized to be therapeutic in the treatment of schizophrenia. Central administration of NT does in fact cause antipsychotic-like effects (Jolicoeur et al., 1993), and therefore a great interest exists for developing NT-mimetics for the treatment of neuropsychiatric diseases, such as schizophrenia.

#### The potential role of NT as an antipsychotic drug

Several lines of evidence suggest that antipsychotics, the traditional treatment for schizophrenia, act through the induction of endogenous NT (Kinkead et al., 1999): (1) all clinically effective antipsychotic drugs affect in some way the NT system of rats. The therapeutic potency of typical APDs is derived mainly from their ability to antagonize DA D_2_ receptors with high affinity (Creese et al., 1976). Typical antipsychotics are associated with extrapyramidal side effects (ESP) characterized by movement disorders including tardive dyskinesia, dystonic reactions, and Parkinsonism. Alternatively, it is suggested that atypical APDs have a higher affinity for 5-HT_2A_ relative to DA D_2_ receptors (Meltzer et al., 1989; Nordstrom et al., 1995; Kapur et al., 1998; Gefvert et al., 2001). They are characterized by enhanced antipsychotic efficacy and lower risk of EPS (Meltzer et al., 1989). Both typical and atypical antipsychotics, those associated with little or no EPS, increase NT mRNA in specific regions of the brain after both acute and chronic treatment (Govoni et al., 1980; Merchant et al., 1991, 1992; Merchant and Dorsa, 1993). (2) These effects are selective for drugs with antipsychotic efficacy, and are not seen with other classes of psychotropic drugs. (3) Typical and atypical antipsychotic drugs differentially affect the NT system. Typical antipsychotics act on both the mesolimbic and nigro-striatal NT systems. By contrast, the actions of atypical antipsychotics on NT are thought to be restricted to the mesolimbic system, the region thought to be the site of therapeutic effect in schizophrenia. (4) Centrally administered NT elicits similar behavioral effects as systemically administered atypical antipsychotic drugs. It is hypothesized that the increase in NT neurotransmission in the mesolimbic system mediates the therapeutic effects of APDs, while the increase in NT neurotransmission in the nigrostriatum underlies the propensity to cause ESP (Binder et al., 2001b; Caceda et al., 2006). Interestingly, the pattern of increase in NT mRNA expression, NT tissue content and release (Kinkead et al., 1999) is similar to that of the immediate early genes, c-fos, FosB, and ΔFos B, after acute and chronic administration of typical and atypical APDs (Binder et al., 2001b).

The ability of a compound to inhibit climbing induced by apomorphine (a DA agonist), hyperactivity induced by amphetamine (indirect DA agonist), and to prevent the disruption of prepulse inhibition (PPI) induced by amphetamine [1-(2,5-dimethoxy-4-iodophenyl)-2-aminopropane] (DOI) (5-HT_2A_ agonist), and dizocilpine (non-competitive NMDA antagonist) are tests used to predict antipsychotic-like activity of a drug. These tests also distinguish between typical and atypical antipsychotic-like effects (Merchant and Dorsa, 1993; Arnt et al., 1995) (see review by Geyer and Ellenbroek, 2003). The ability of a compound to block apomorphine-induced climbing over oro-facial stereotypies (sniffing and licking) induced by high doses of apomorphine, suggests atypicality of that compound (Gerhardt et al., 1985).

Neurotensin has been shown to prevent the disruption of amphetamine- and apomorphine-induced PPI (Feifel et al., 1997), as well as attenuate apomorphine-induced climbing (Jolicoeur et al., 1993), and amphetamine- and apomorphine-induced hyperactivity without affecting stereotypy (Jolicoeur et al., 1983). For these reasons it is hypothesized that NT transmission is integral to the mechanism of action of antipsychotic drugs and that NT can serve as an endogenous antipsychotic-like compound (reviewed in Kinkead et al., 1999; Binder et al., 2001b; Kinkead and Nemeroff, 2002).

#### The behavioral effects of neurotensin analogs in animal models of schizophrenia

As discussed above, there is strong evidence to support NT as an endogenous antipsychotic drug. However, NT is easily degraded by peptidases and cannot cross the blood-brain barrier. In an effort to study more easily the effects of NT, our group has developed an extensive series of NT(8–13) analogs that can be delivered peripherally, and that elicit effects similar to centrally administered NT. These NT analogs have demonstrated antipsychotic-like activity similar to endogenous NT as determined by behavioral tests in rats. Most of our work has been with NT69L and NT79. NT69L binds human NTS1 and human NTS2 with high affinity. By contrast, NT79 preferentially binds to NTS2, as its binding affinity for the human NTS2 is approximately 25-fold higher than that for human NTS1. NT69L (Cusack et al., 2000), NT79 (Boules et al., 2010), and another analog NT77L (Boules et al., 2001c) are each modified from NT at amino acid positions located in the C-terminal 8–13 sequence. Additionally, PD149163, which is a reduced amide bond NT(8–13) mimetic shows antipsychotic-like activity.

PD149163 has strong affinity for NTRs (*K*_i_ = 31.2 nM in newborn mouse brain membranes) and improved metabolic stability (Wustrow et al., 1995), a factor that promotes central activity after systemic administration (Banks et al., 1995).

Table 1 shows the structures of some NT analogs.

**Table 1:** **Structures of NT analogs**.

	1	2	3	4	5	6	7	8	9	10	11	12	13
NT	*p*-Glu	l-Leu	l-Tyr	l-Glu	l-Asn	l-Lys	l-Pro	l-Arg	l-Arg	l-Pro	l-Tyr	l-Ile	l-Leu
NT69L	–	–	–	–	–	–	–	*N*-methyl-Arg	l-Lys	l-Pro	l-neo-Trp	Tert-Leu-	l-Leu
NT72									d-Lys	l-Pto	l-neo-trp	tert-	l-Leu
NT77L	–	–	–	–	–	–	–	l-Arg	d-Orn	l-Pro	l-neo-Trp	tert-Leu	l-Leu
NT79	–	–	–	–	–	–	–	*N*-methyl-Arg	l-Arg	l-Pro	d-3,1-Nal	tert-Leu	l-Leu
PD149163								H-Lys-psi[CH2NH]	Lys	Pro	Trp	Tle	Leu-Oet

NT69L elicits similar neurochemical and behavioral effects as endogenous NT (for review, see Boules et al., 2003a). Intraperitoneal delivery of NT69L selectively inhibits stereotyped apomorphine-induced climbing at an ED_50_ of 16 μg/kg, without affecting licking or sniffing at any dose given (Cusack et al., 2000). Additionally, NT69L does not cause catalepsy (muscle rigidity) at any dose given, but reverses haloperidol-induced catalepsy with an ED_50_ of 0.2 mg/kg (Cusack et al., 2000). Catalepsy in rats has been used as a predictor of EPS potential of APDs in humans.

NT69L significantly increases both amphetamine- and dizocilpine-induced decreases in PPI with acute administration with an ED_50_ of 0.08 and 0.05 mg/kg respectively (Shilling et al., 2003; Boules et al., 2010), as well as with chronic administration (Briody et al., 2010). Additionally, NT69L blocks PCP-induced hyperactivity (Li et al., 2010a), a test that may reflect both positive and negative symptoms of schizophrenia (Snyder, 1980; Javitt and Zukin, 1991). These results demonstrate that NT69L has properties similar to those of atypical APDs.

NT77 is also thought to have antipsychotic-like properties, although less potently than NT69L, based on similar rat behavioral studies (Boules et al., 2001c). NT77L selectively blocks apomorphine-induced climbing with an ED_50_ of 5.6 mg/kg without affecting sniffing or licking behavior at any dose. NT77L moderately prevents/reverses the cataleptic effect of haloperidol (ED_50_ 5.6 mg/kg) while it does not cause catalepsy itself at any dose. The effects of NT77L on PPI have yet to be tested.

Like NT69L and NT77L, NT79 also selectively blocks apomorphine-induced climbing without affecting sniffing or licking behavior at any dose. Similarly, NT79 significantly blocks amphetamine-induced hyperactivity as well as prevents the disruption of PPI induced by both amphetamine and DOI (Boules et al., 2010). PD149163 antagonizes the reduction in PPI of the rat startle reflex produced by amphetamine and dizocilpine (Feifel et al., 1999), blocks the disruption in PPI induced by the 5-HT_2A_ agonist DOI (Feifel et al., 2003), and inhibits conditioned avoidance responding, which is a highly validated test for screening APD in rats, without causing catalepsy (Holly et al., 2011). These analogs show promise for use in the treatment of schizophrenic symptoms with a lower potential for adverse side effects. The antipsychotic effects of NT analogs are summarized in Table 2.

**Table 2:** **Antipsychotic effects of NT analogs**.

Analog	Apomorphine-induced climbing	Reversal of Haloperidol-induced catalepsy	Amphetamine-induced decrease in PPI and hyperactivity	Dizocilpine/DOI-induced decrease in PPI	PCP-induced hyperactivity
NT69L	Y^1^	Y^2^	Y^3^	Y^4^	Y^5^
NT77L	Y^6^	Y^6^			
NT79	Y^7^	Y^7^	Y^7^	Y^7^	
PD149163			Y^8,9^	Y^10^	

*Y = has an effect*.

*^1^Cusack et al. (2000), Mechanic et al. (2009); ^2^Cusack et al. (2000); ^3^Boules et al. (2001a), Shilling et al. (2003), Briody et al. (2010); ^4^Shilling et al. (2003), Secchi et al. (2009); ^5^Li et al. (2010a); ^6^Boules et al. (2001c); ^7^Boules et al. (2010); ^8^Feifel et al. (2008); ^9^Feifel et al. (1999), Shilling et al. (2004); ^10^Feifel et al. (1999), Feifel et al. (2003), Shilling et al. (2004)*.

Studies with the use of knockout mice lacking NT or NTR subtypes suggest a role for NTS1 in the antipsychotic-like effect of NT. NT knockout mice have reduced PPI and are not sensitive to the PPI-disrupting effects of amphetamine as compared to wild type mice. These data indicate the importance of endogenous NT in the effects of amphetamine on PPI (Mechanic et al., 2009). Additionally, the use of NTS1- and NTS2-knockout (NTS1^−/−^ and NTS2^−/−^) mice revealed hyper-dopaminergic state in the NTS1^−/−^ mice similar to the excessive striatal DA activity reported in schizophrenia (Liang et al., 2010). Furthermore, the NTS1^−/−^ show changes in behavior, prefrontal cortex neurotransmitters, and protein expression that are similar to wild type mice treated with the psychomimetic PCP, an animal model for schizophrenia (Li et al., 2010c). The involvement of NTS1 in the antipsychotic-like effect of NT has been further demonstrated by the use of NT agonists. Administration of the NT agonists, NT69L and NT-2, reversed apomorphine-induced climbing in wild type but not in NTS1^−/−^ mice (Mechanic et al., 2009). Likewise, PD149163 significantly facilitates PPI and decreases the acoustic startle response in wild type but not in NTS1^−/−^ mice (Feifel et al., 2010b).

### Pain

#### NT and pain

Neurotensin and NTRs modulate nociception at several different levels (Dobner, 2005). In fact, on a molar basis, NT is more potent than is morphine as an antinociceptive agent (Nemeroff et al., 1979; al-Rodhan et al., 1991). Central administration of NT induces an analgesic response in both the hot plate (HP) and acetic acid-induced writhing tests in rats (Clineschmidt and McGuffin, 1977; Clineschmidt et al., 1979, 1982). NT also produces a long-lasting antinociceptive effect in the tail-flick (TF) assay following intra rostroventral medulla (RVM) administration (Fang et al., 1987). These results indicate that NT can influence nociceptive transmission at several different points in the descending pain modulatory circuitry in the brain. There is also limited evidence that NT may affect nociception transmission directly in the spinal cord (Dobner, 2006). Intrathecal NT administration increased both HP and aversive (hypertonic saline) response latencies, but had no significant analgesic effect in the TF (Hylden and Wilcox, 1983). In addition, intrathecal injection of either NT or one of our novel NT(8–13) into the spinal cord of rats modifies pain perception in a rodent model of persistent (Roussy et al., 2006) and of neuropathic pain (Dansereau et al., 2006).

#### NT receptor subtypes and pain

The effect of NT on pain modulation is dose-dependent and receptor-selective (Urban and Smith, 1993). NT not only inhibits but also facilitates pain transmission in a dose-dependent manner. High doses (nanomolar range) of the peptide injected into the RVM have an antinociceptive effect as measured by the TF latency (TFL) in response to heat stimulus. In contrast, lower doses (picomolar range) have been shown to reduce latencies in the HP and TF tests, facilitate spinal nociception response, and increase the visceromotor response to noxious heat and visceral stimulation respectively (Urban and Smith, 1993, 1994).

The basis of the opposing actions of NT and its dose-dependent modulation is most probably due to separate and distinct NTR subtype with varying affinity for the peptide (Smith et al., 1997), as well as the involvement of separate and distinct neuronal pathways that modulate pain at the spinal level (Smith et al., 1997). Evidence suggests that both NTS1 and NTS2 mediate the antinociceptive effects of NT, depending upon the antinociceptive test and possibly, the species of rodent used. NTS1 and NTS2 modulate pain-induced behavioral responses by acting on distinct spinal and/or supra-spinal neural circuits (Roussy et al., 2008). Contradictory results have been reported regarding the NTR subtype mediating the antinociceptive effects of NT.

##### NTS1 and pain

Reports on mice lacking the NTS1 gene reveal that NT and NT analogs fail to induce antinociception in the HP test (Pettibone et al., 2002). Consistent with the knockout mice studies, our group showed that the inhibition of NTS1 synthesis with the use of antisense peptide nucleic acids (PNAs) targeting the NTS1 gene also results in loss of the analgesic properties of NT in the HP test (Tyler et al., 1998b), results that implicate NTS1 in thermal analgesia. Conversely, other evidence that argues against the involvement of NTS1 in the analgesic effect of NT has been reported and summarized by Dubuc et al. (1999a). Thus, as summarized by this group, the analgesic effects of NT are not antagonized by the NTS1-selective antagonist SR48692 (Dubuc et al., 1994), the binding affinity of NT analogs to NTS1 does not correlate with their analgesic effects (Labbe-Jullie et al., 1994), and the administration of antisense oligonucleotides targeted to NTS1 does not reduce NT-induced analgesia in the writhing test in mice (Dubuc et al., 1999b). With respect to formalin-induced pain, Roussy et al. (2010) concluded that NTS1 is important for modulation of persistent pain following systemic administration of morphine in NTS1^−/−^ mice.

##### NTS2 and pain

NTS2 has also been implicated in the analgesic effects of NT (Dubuc et al., 1999b; Remaury et al., 2002; Yamauchi et al., 2003; Maeno et al., 2004; Sarret et al., 2005; Bredeloux et al., 2006). Dubuc et al. showed, with the use of NTS2 oligonucleotides, that NTS2 plays an important role in the writhing test. These data were supported by a close correlation between NT analog binding affinity at NTS2 and potency of the analog-induced analgesia in mice (Dubuc et al., 1999b). Maeno et al. (2004) working with mice, suggested that NTS2 plays an important role in thermal nociception. The role of NTS2 in mediating NT-induced analgesia has been based on observations using NTS2 antisense oligodeoxynucleotides and the NTS2 ligand levocabastine (Bredeloux et al., 2006). NTS2 has also been implicated in mediating visceral antinociception (Dubuc et al., 1999a).

#### NT analogs and pain

As mentioned, NT must be administered directly into the brain to exert its effect since it gets degraded by peptidases (Tyler-McMahon et al., 2000a; Boules et al., 2005). Our laboratory has been testing our brain-penetrating stable analogs of NT(8–13) in several animal models of pain.

Studies by our group and others show that NT analogs are effective in treating thermal, visceral (acetic acid-induced writhing), and persistent inflammatory (formalin-induced) pain (Tyler-McMahon et al., 2000b; Bredeloux et al., 2008; Boules et al., 2009, 2010; Mechanic et al., 2009). However, as previously stated, evidence suggests that the analgesic efficacy of NT analogs varies with their selectivity for NTS1 and NTS2, the pain model, and, probably, animal species.

Intrathecal injection of the NTS1-selective agonist, PD149163, and the non-selective NT agonist NT69L significantly reduced pain-evoked responses during the inflammatory phase of the formalin test (Roussy et al., 2008). The same analogs also produced potent antiallodynic and antihyperalgesic effects in nerve injured rats, a model of neuropathic pain (Guillemette et al., 2012). Systemic injection of NT analogs NT69L, NT72 (NTS1-selective), and NT77 causes analgesia in the HP test (Tyler et al., 1998a, 1999; Boules et al., 2001c; Smith et al., 2011) with synergy to morphine (Boules et al., 2009). Additionally, NT69L and NT72, significantly reduce acetic acid-induced writhing (Smith et al., 2012).

Supporting the role of NTS2 in antinociception, intrathecal injection of NTS2 agonists levocabastine and JMV431 significantly inhibit the aversive behavior induced by formalin (Roussy et al., 2009) and induced dose-dependent antinociceptive response in the TF test (Sarret et al., 2005).

Interestingly, the NTS2-selective analog, NT79, is ineffective in reducing thermal pain, but blocks acetic acid-induced writhing in rats (Boules et al., 2010), without causing tolerance to its analgesic effects (Smith et al., 2012). It is also interesting that NT79 does not cause hypothermia. Since the NTS1-selective (NT72), and non-selective (NT69L) NT agonists attenuate visceral nociception, it is suggested that both NTR subtypes are involved in mediating visceral analgesia and their roles appear to be NT analog dependent (Smith et al., 2012). Additionally, the NTS2-selective analog, NT79, reduces formalin-induced pain and does so in synergy with morphine (Boules et al., 2011a). Further evidence for the involvement of NTS2 in reducing persistent pain comes with the use of knockout mice. Lafrance et al. (2010) demonstrated that mice lacking NTS2 exhibit significantly lower stress-induced analgesia following cold-water swim stress as compared to their wild type littermates. Table 3 summarizes the analgesic effects of NT analogs.

**Table 3:** **Analgesic effects of NT analogs**.

Analog	Hot plate	Writhing	Formalin	NP
NT69L	Y^1^	Y^2^	Y^3^	Y^4^
NT72	Y^2^	Y^2^		
NT77	Y^5^			
NT79	N^6^	Y^6^	Y^7^	Y^8^
PD149163			Y^9^	Y^4^

*Y = has an effect; N = has no effect*.

*^1^Tyler-McMahon et al. (2000b), Boules et al. (2009), Smith et al. (2011); ^2^Smith et al. (2012); ^3^Roussy et al. (2008), Roussy et al. (2009); ^4^Guillemette et al. (2012); ^5^Boules et al. (2001c); ^6^Boules et al. (2010); ^7^Boules et al. (2011a); ^8^Boules et al. (2012); ^9^Roussy et al. (2008), Roussy et al. (2009)*.

#### Analgesic synergy between NT and morphine

Morphine is a μ-opioid receptor agonist that is widely used for the treatment of many types of chronic pain. However, morphine and other opioids are usually associated with some serious side effects. Additionally, tolerance develops to their analgesic effects, but not to all the side effects, and this tolerance requires dosage increases over time, to attain a consistent level of analgesia. Increasing morphine dosage increases the potential for serious undesired side effects, such as respiratory depression.

Neurotensin exerts a potent μ-opioid-independent, antinociceptive effects in a variety of analgesic screening tests, including tail-flick, HP, and writhing induced by acetic acid (Clineschmidt et al., 1979, 1982; al-Rodhan et al., 1991; Wustrow et al., 1995; Sarhan et al., 1997). Both NTS2 and μ-opioid receptors have been found in the same brain structures involved in pain perception (Basbaum and Fields, 1984; Asselin et al., 2001) and neurotensinergic system seems to play an important role in the non-opioid form of stress-induced analgesia (Seta et al., 2001; Gui et al., 2004; Lafrance et al., 2010).

The functional analgesic interaction between endogenous NT and the opioid system has been further illustrated by Tershner and Helmstetter. These authors show that the antinociception induced by the μ-opioid receptor activation in the amygdala is partly dependent on the recruitment of NTRs in the ventral PAG (Tershner and Helmstetter, 2000). Additionally, mice rendered tolerant to morphine showed a reduced analgesic effect to NT (Luttinger et al., 1983) and morphine analgesia was considerably reduced in NTS1-deficient mice. These data indicate that the NTS1 actively participates in μ-opioid analgesia (Roussy et al., 2010). Conversely, opioid receptor antagonists do not block NT-mediated antinociception (Clineschmidt et al., 1982; al-Rodhan et al., 1991), but the perfusion of morphine in the PAG increases the extracellular levels of NT-ir in a naloxone-dependent manner (Stiller et al., 1997).

The use of adjuncts such as the NMDA receptor antagonist ketamine in combination with opioids to enhance their efficacy at low doses, and attenuate the occurrence of tolerance has been reported (Lutfy et al., 1996; Nishiyama, 2000). However, serious motor impairment is observed at doses of ketamine that are antinociceptive in the rat and in human, and ketamine can be psychotomimetic. Additionally, long-term exposure of ketamine on spinal tissue leads to tissue necrosis (Vranken et al., 2005). Studies in our laboratory show analgesic synergism between NT analogs and morphine in the use of HP (Boules et al., 2009), in acetic acid-induced writhing, and in formalin-induced pain (Boules et al., 2011a). These studies provide novel potential use for NT analogs in combination with morphine (and perhaps other opioids) to improve the pharmacological treatment of pain while minimizing specific adverse effects of each of the drugs at a higher dose.

### NT and psychostimulant abuse

Psychostimulants, including nicotine, amphetamine, and cocaine, are drugs that may be employed to improve cognitive and motor function, but can be highly addictive. The mesocorticolimbic system, to which NT colocalizes with DA and other neurotransmitters, provides the anatomic substrate for psychostimulant dependence and craving (McBride et al., 1999; Di Chiara, 2000). NT acts as a neurotransmitter as well as a modulator of DA and other monoamine neurotransmitter systems and has been linked through several lines of evidence to psychostimulant effects (Richelson et al., 2003).

Behavioral sensitization to psychostimulants, an animal model of addiction, is a process by which repeated administration of the same dose of a drug produces increasing degrees of locomotor effects (Domino, 2001). This process is thought to be due to changes in the NA. Initiation of sensitization is associated with changes in the NA shell, and maintenance of sensitization with changes in the NA core (Iyaniwura et al., 2001; Balfour, 2004). Behavioral sensitization models human acquisition of psychostimulant addiction and risk of relapse (Miller et al., 2001; De Vries et al., 2002).

Locomotor sensitization depends on activation of the mesolimbic DA system with additional long-term influences on glutamate, GABA, κ-opioid, and other neurotransmitter systems (Pierce and Kalivas, 1997; Hahn et al., 2000). DA neurons originating in the VTA and projecting to the shell and core of the NA lead to the initiation and expression of sensitization, respectively (Pierce and Kalivas, 1997). NT colocalizes with DA neurons and acts as a modulator of DA effects (Binder et al., 2001a). When injected into the VTA, NT causes hyperactivity and DA release in the NA, similar to the effects of psychostimulants (Kalivas and Duffy, 1990; Kalivas, 1994). However, NT injected into the NA reduces the response to psychostimulants, an effect similar to that of brain-penetrating NT analogs given extracranially (Ervin et al., 1981; Robledo et al., 1993; Richelson et al., 2003).

The NT agonist NT69L, given intraperitoneally, blocks the acute locomotor effects of cocaine and d-amphetamine (Boules et al., 2001a), and blocks both the initiation and expression of sensitization to nicotine after subcutaneous administration (Fredrickson et al., 2003a,b). This is one line of evidence suggesting NT agonists may be effective for treatment of nicotine, amphetamine, and cocaine addiction. Curiously, the NT antagonist SR48692 when administered chronically decreased locomotor sensitization to cocaine (Felszeghy et al., 2007).

How then can a NTR agonist and a NTR antagonist have similar effects in this particular animal model of addiction? Although further research is needed, several lines of evidence bear on this question. Repeated doses of the NT antagonist were required to produce the intended effect on sensitization. Classically, chronic receptor blockade can lead to up-regulation of the receptor, thus making the agonist more effective. Chronic blockade of the receptors may also lead to increased NT synthesis and release. Another approach to this question is with animals lacking NTRs. Studies with SR48692 would predict that mice lacking NTS1 would not sensitize to psychostimulants. However, such null mice are more sensitive to an acute injection of d-amphetamine and had an enhanced response to the sensitizing effects of this psychostimulant as compared to wild type mice (Boules et al., 2006).

The NT agonist NT69L attenuates intravenous (IV) self-administration of nicotine in rats (Boules et al., 2011b). Once animals acquired stable responding to nicotine they were pretreated with either NT69L or saline. Pretreatment with NT69L attenuated nicotine self-infusion under FR1 (fixed ratio of 1) and FR5 schedules of reinforcement. Control rats that were response-independent “yoked” as well as rats that self-infused saline showed minimal responses, indicating that nicotine served as a reinforcer. The stimulant and reinforcing effects of nicotine are attributed to stimulation of the mesolimbic DA system (Stein et al., 1998; Di Chiara, 2000; George et al., 2000). In this study, nicotine self-infusion increased TH, DA D_1_, and DA D_2_ receptor mRNA in the VTA, consistent with increased burst firing. NT69L antagonized these effects. In the PFC, an area implicated with learning, NT69L increased TH and DA D_1_ mRNA. TH and DA D_1_ mRNA levels were also increased in the striatum in response to the NT agonist. Taken together these results show that the NT agonist NT69L attenuated nicotine self-administration and suggest the drug would reduce craving and withdrawal symptoms (for example, cognitive complaints that are common during nicotine withdrawal).

Is there abuse potential for NT agonists? Although NT mimics the effects of psychostimulants when injected directly into the VTA, it blocks certain stimulant effects when injected into the NA. NT analogs given extracranially (native NT is degraded by peptidases when given peripherally) also block locomotor effects of stimulants, and attenuate nicotine self-administration in rats. Rats do not self administer NT. Therefore, the preponderance of evidence argues against an abuse or addiction potential for NT analogs. This conclusion is supported by data in rhesus monkeys; the animals did not self-infuse NT69L (Fantegrossi et al., 2005).

Although questions remain, a growing body of data supports a key role for NT in regulation of responses to psychostimulants. To the extent animal models are predictive, and NT agonists show promise for the treatment of human addiction. The relative contributions of NTS1 and NTS2 receptors in these models have not been extensively explored, and further research may lead to development of agonists which retain efficacy with fewer side effects such as hypothermia and hypotension.

### NT and Parkinson’s disease

Studies show that plasma NT concentrations are consistently higher in PD patients as compared to controls and untreated as compared to treated patients (Schimpff et al., 2001). PD patients also have a twofold increase in NT content in both zona compacta and zona reticulate of the SN compared to that for controls (Fernandez et al., 1995). Brains from PD patients have fewer dopaminergic neurons and very low expression of NTS1 (Yamada and Richelson, 1995). Similar results were reported in an animal model for PD, where MPTP treated mice had significantly lower [^3^H] NT and [^3^H] mazindol binding in the striatum and SN, results indicating severe reduction in NTRs and DA uptake sites respectively. These data suggest that the dysfunction in NTRs may be involved in the degradation processes causing PD. Caceda et al. (2006) suggested that the increase in striatal and nigral NT tissue concentrations, as well as in CSF and plasma levels may be due either to a compensatory mechanism for the loss of DA neurons to preserve motor function and/or to a dysregulation of NT neurotransmission on striatal output favoring the striatopallidal pathway (Caceda et al., 2006). Injection of NT resulted in a dose related attenuation of muscular rigidity and tremors caused by bilateral injection of the neurotoxin 6-hydroxydopamine in the medial forebrain bundle (Jolicoeur et al., 1991). In addition, we showed that injection of the NT agonist, NT69L, attenuated the amphetamine and apomorphine rotatory behavior caused by the unilateral injection of the same drug in the nigro-striatal pathway (Boules et al., 2001b).

## Potential Side Effects of NT Agonists

### Hypothermia

Neurotensin (Bissette et al., 1976) and NT agonists (Tyler-McMahon et al., 2000b; Boules et al., 2001c, 2003a; Feifel et al., 2010a) elicit hypothermia in rodents. Hypothermia is mediated by NTS1. This has been established with the use of NTS1^−/−^ and NTS2^−/−^ mice (Remaury et al., 2002; Mechanic et al., 2009). Additionally, NTS2-selective analogs do not induce hypothermia (Boules et al., 2010), results that provide further proof for the involvement of NTS1 receptor subtype in mediating hypothermia. Interestingly, studies show potential therapeutic use for the NT agonist-induced hypothermia. The administration of the NT agonist ABS-201 immediately or up to 60 min after stroke attack significantly reduced infarct formation and brain cell death in an animal model of focal ischemia (Choi et al., 2012). In addition, it was effective in promoting long-term functional recovery in post-stroke animals (Choi et al., 2012). Similar studies on regulated hypothermia induced by the NT agonists reduce oxidative stress in the brain during reperfusion from asphyxial cardiac arrest (Katz et al., 2004a). Also, lowering body temperature with a NT agonist provided a better neurologic outcome than brief external cooling in a rat model of near drowning (Katz et al., 2004b). Consistent with these results, hypothermia induced by the NT analog JMV-449 had a neuroprotective effect in a mouse model of permanent distal middle cerebral artery occlusion (Torup et al., 2003).

### Hypotension

Intracerebroventricular (ICV) or IV injections of NT induces a dose-dependent drop in arterial blood pressure in anesthetized rats. The drop in blood pressure is of rapid onset (30–60 s) and of short duration (1–4 min) (Rioux et al., 1981). Interestingly, the hypotensive effect of NT is not accompanied by any alteration in cardiac actions (Rosell et al., 1976; Rioux et al., 1981).

### Development of tolerance

Animal studies show that tolerance develops to some but not all the effects of NT analogs. Tolerance develops to the hypothermia, thermal analgesia, and anticataleptic effect of NTS1 agonists (Boules et al., 2003b). Conversely, tolerance does not occur to the NT agonist’s effects on the reversal of amphetamine- and cocaine-induced locomotor activity (Boules et al., 2003b; Hadden et al., 2005; Feifel et al., 2008), the reversal of apomorphine-induced climbing (Boules et al., 2003b), and the reversal of amphetamine or DOI-induced disruption of PPI (Feifel et al., 2007; Briody et al., 2010).

Interestingly, NTS2 seem to play an important role in the development of tolerance of NT69L-mediated hypothermia and thermal analgesia (Smith et al., 2011), while tolerance to its analgesic and anticataleptic effects the NTS2 analog NT79 does not occur (Boules et al., 2010).

Thus, the development of tolerance depends on the NT analog, their selectivity to the NTR subtypes, the paradigm being tested and the dosing regimen (Wang et al., 2005; Feifel et al., 2008).

## Conclusion

The association of NT with several neurotransmitter and endocrine systems suggests its involvement in a wide range of physiologic processes throughout the body and implicates it in the pathology of many neuropsychiatric diseases. Furthermore, the development of NT analogs that can be administered systemically may provide therapy for the treatment of several disorders including schizophrenia, pain, and psychostimulant abuse. Additionally, the use of NT analogs and genetically modified animals will allow further understanding of the molecular role of NT in health and disease.

## Conflict of Interest Statement

Drs Richelson, Fredrickson, and Boules have a U.S. Patent # 7,087,575, August 8, 2006. “Treating the Effect of Nicotine: Elliott Richelson, Paul Fredrickson, Mona Boules.”
